# Propagatory dynamics of nucleus-acoustic waves excited in gyrogravitating degenerate quantum plasmas electrostatically confined in curved geometry

**DOI:** 10.1038/s41598-021-98543-2

**Published:** 2021-09-27

**Authors:** Sayanti Dasgupta, Pralay Kumar Karmakar

**Affiliations:** Department of Physics, Tezpur University, Napaam, Tezpur, Assam 784028 India

**Keywords:** Astronomy and planetary science, Physics

## Abstract

A theoretic model to investigate the dynamics of the longitudinal nucleus-acoustic waves (NAWs) in gyrogravitating electrostatically confined degenerate quantum plasma (DQP) system in spherically symmetric geometry is constructed. The model setup consists of non-degenerate heavy nuclear species (HNS), lighter nuclear species (LNS), and quantum degenerate electronic species (DES). It specifically considers the influences of the Bohm potential, Coriolis rotation, viscoelasticity, and electrostatic confinement pressure (ECP, scaling quadratically in density). A standard normal spherical mode analysis gives a generalized dispersion relation (septic). It highlights the dependency of various atypical instability response on the equilibrium plasma parameters. A numerical illustrative platform portrays that the relative nuclear charge-to-mass coupling parameter ($\beta$) acts as a destabilizing agency and the heavy-to-light nuclear charge density ratio ($\mu$) acts as a stabilizing agency in both the non-relativistic (NR) and ultra-relativistic (UR) limits. Another interesting conjuncture is that the Coriolis rotation introduces a destabilizing influence on the system in both the limits. The progressive analysis presented herein has correlations and consistencies in the dynamic growth backdrop of various compact astro objects and their circumvent atmospheres, such as white dwarfs, neutron stars, etc.

## Introduction

The area of quantum plasmas is one of the most rapidly evolving research fields due to its large scale interdisciplinary scope of potential applications extensively ranging from nanoscales to astrocosmical scales of space and time^1–4^. Such quantum plasmas are widely characterized by very high particle number density (~ $10^{29}$–$10^{36}$ m^−3^), and extremely low temperature scales (T ~ T_F_), in contrast to the conventional classical plasmas, usually characterized with low density, and high temperature^1,2,4^. Quantum degenerate matter is found to exist naturalistically in diversified compact astrophysical objects, such as stellar cores, white dwarfs, black dwarfs, neutron stars, and interiors of giant planets in the solar system^4,5^, where the constitutive particles form a degenerate system under the extreme conditions of high density and low temperature^4^. In quantum systems, the mean interparticle distance (mean free path) becomes comparable to or smaller than the de-Broglie wavelength associated with the constituent particles. The de-Broglie wavelength gives a rough representation of the spatial expansion of the particle wave function, indicating that the electrons will exhibit the quantum behaviour with much more prominence as compared to the ions, on account of their large mass difference^6^.

The degeneracy of these extraordinarily dense quantum plasmas can be traced back to the combined action of the Pauli exclusion principle and the Heisenberg uncertainty principle^7,8^. The particles are highly uncertain in their momenta, as a result of the high compression by virtue of their location in an extremely confined space. The particles travel very fast, in spite of being extremely cold, thus giving rise to a very high pressure, termed as the degeneracy pressure^9,10^. A general expression of this pressure in the case of electron fluid is given with the help of the polytropic pressure law given as^9,10^: $P_{e} \, = \,K_{e} \,n_{e}^{{\gamma_{e} }} \,$; where, the polytropicity constant, $K_{e} = {{3\lambda_{c} \hbar c} \mathord{\left/ {\vphantom {{3\lambda_{c} \hbar c} 5}} \right. \kern-\nulldelimiterspace} 5}$ (with $\lambda_{c} = {{\pi \,\hbar } \mathord{\left/ {\vphantom {{\pi \,\hbar } {m_{e} c}}} \right. \kern-\nulldelimiterspace} {m_{e} c}}$), and the polytropicity exponent, $\gamma_{e} = {5 \mathord{\left/ {\vphantom {5 3}} \right. \kern-\nulldelimiterspace} 3}$, for the NR limit. This is in contrast with the UR corresponding counterparts, given as, $\gamma_{e} = {4 \mathord{\left/ {\vphantom {4 3}} \right. \kern-\nulldelimiterspace} 3}$ and $K_{e} = {{3\hbar c} \mathord{\left/ {\vphantom {{3\hbar c} 4}} \right. \kern-\nulldelimiterspace} 4}$ ^9,10^.

It has been confirmed with the help of astronomical observations that the white dwarfs basically constitute the quantum mechanical tiny DES, weakly coupled LNS (hydrogen (H) and helium (He)), and strongly coupled HNS (carbon (C) and oxygen (O))^11–13^. The electrons are relativistically degenerate within the inner core and non-relativistically degenerate in the outer mantle of the white dwarf stars^1,2^. A significantly large number of investigations have been made to analyze mostly the various nonlinear structures associated with the nucleus-acoustic wave (NAW) mode, in both the planar and nonplanar geometry^14–18^. Such NAWs are the propagatory longitudinal oscillations triggered due to the interplay between the inertia (by heavy nuclei) and the non-thermal elasticity (by degenerate electrons). The dynamics of the corresponding nucleus-acoustic shock structures formed in strongly coupled self-gravitating DQPs has been analyzed in the recent past^14^. Again, reductive perturbation method has been employed to study the naturalistic features of the nucleus-acoustic double layers and solitary waves in magnetized DQP^8^. Heavy nucleus-acoustic spherical solitons in a self-gravitating degenerate quantum plasma have been theoretically investigated^15^. The basic properties of nucleus-acoustic shock structures have also been investigated in both the planar and nonplanar geometrical construct with the help of the modified Burgers equation^7^. Besides, the existence of the NAWs in a cold DQP system has also been addressed^16^.

The cubic nonlinear Schrodinger (NLS) equation has been used to investigate the nonlinear dynamics of the heavy NAWs and nucleus-acoustic envelope solitons for both modulationally stable and unstable regimes^17,18^. The influential role of the oblique magnetic field has also been analyzed in the formation of the nucleus-acoustic solitary structures^19^. Again, the properties of the nucleus-acoustic solitary-shock waves originating in white dwarf system have been theoretically analyzed^20,21^. It has also been found that the nucleus-acoustic eigen modes exist with positive electrostatic and negative self-gravitational potential in self-gravitating degenerate DQP^22^. A systematic study of the excitation, propagation and stability of the same has been done in both the linear and nonlinear regimes^1,2^. The basic properties of the subsonic and supersonic nucleus-acoustic shock structures have been studied by employing the pseudo-potential approach^23^. It can be clearly seen that their excitation and propagatory dynamics in a gyrogravitating degenerate electrostatically confined quantum plasma system has hitherto been remaining an unaddressed problem, yet to be investigated both theoretically and numerically.

In our semi-analytic theoretic study, we investigate the excitation and propagatory dynamics of the NAWs in the linear regime in a gyrogravitating DQP system in a spherically symmetric geometrical configuration. We consider the conjoint complex effects of the Bohm potential, Coriolis rotation, ECP^24^, viscoelasticity^25,26^, and self-gravity^25,26^ simultaneously in three-component DQP system. The main novelty of the proposed model lies in the consideration of the ECP, Coriolis rotation, and the multi-component degenerate plasma system, which are indeed found to exist in diversified compact astroobjects, such as white dwarfs^11–13^. Out of all these well-known factors, it is only the ECP which scales quadratically with number density, which is included in such situations for the first time. This nonlinear quadratic contribution is much larger than the linear thermal pressure on the population density^24^. The system comprises of strongly coupled HNS (classical), weakly coupled LNS (classical), and NR-UR DES (quantum). It is needless to mention further that the proposed model very closely mimics the compact environment of the white dwarfs. The importance of the ECP term can be realized from the fact that in white dwarfs, the heavy and LNS are usually confined in a cloud within the core by their auto-generated electric fields. In such circumstances, there are large-scale mean electric fields present within the system, contributing appreciably to the resultant pressure associated with both the heavy and the LNS. The inclusion of the Coriolis rotation makes it more realistically resemble with the gyrogravitating white dwarf environments^27,28^. It may be worth mentioning that the angular momentum associated with the white dwarfs has a primordial origin in the life of the stars. It plays a significant role to boost their modified phase transitions in the Hertzsprung–Russel diagram^29^ during the numerous evolutionary stages leading to their ultimate diversified fates^27,28^. The simultaneous realistic effects considered here are mainly applicable to the rapidly rotating collapsing white dwarfs^30^ and the viscous evolution of remnants of white dwarf mergers, leading to the detonation of their helium (He) envelopes^31^. Thus, the proposed model should have extensive applicability to demonstrate the realistic astronomical modal excitation dynamics in dwarf family stars, other degenerate stars, and their internal environments in correlative consistency with the previously predicted astronomical stability observations^30–32^.

## Physical model and formalism

We consider a gyrogravitating degenerate electrostatically confined quantum plasma system consisting of strongly coupled HNS (classical), weakly coupled LNS (classical), NR-UR DES (non-classical) in a curved (nonplanar) geometry. The dynamics of the complex plasma system is modelled with the help of the generalized hydrodynamic (GH) formalism under a quasi-classic approximation of spherically symmetric geometry free from polar and azimuthal counterparts. The main advantage of the assumed spherical symmetry is in the geometric reduction of the three-dimensional problem into a one-dimensional radial one for the sake of analytic simplicity. The practical realization of such a correlated physical plasma system could be achieved in the interiors of white dwarf stars, particularly the C-O white dwarfs, having the asymptotic mass scaling in the range $0.25M_{\Theta } \, < \,\,M\, < \,\,8M_{\Theta }$; where, $M_{\Theta } = 1.989\, \times 10^{33}$ g is the mass of the Sun^33^.

The basic set of the governing equations include the flux conservation continuity equation, force-balancing momentum equation, and supplementary equation of state^1,2,7,24^. It is systematically closed with the gravitational and electrostatic Poisson equations depicting the potential distributions arising from the heterogeneous density fields^1,2,7^.

The dynamics of the DES is accordingly described with the help of the continuity equation for flux density conservation, momentum equation for force density conservation, and equation of macroscopic state for the sensible pressure-density correlation in the customary notations^1,2,7,9,10^ given respectively as

1 $$\frac{{\partial n_{e} }}{\partial t} + \frac{1}{{r^{2} }}\frac{\partial }{\partial r}\left( {r^{2} n_{e} u_{e} } \right) = 0\,,$$

2 $$e\,\frac{\partial \,\Phi }{{\partial \,r}}\, - \,\frac{1}{{n_{e} }}\,\frac{{\partial \,P_{e} }}{\partial \,r}\, - \,\frac{{\hbar^{2} }}{{2\,m_{e} }}\,\frac{\partial }{\partial \,r}\,\frac{1}{{\sqrt {n_{e} } }}\,\left[ {\frac{1}{{r^{2} }}\,\frac{\partial }{\partial \,r}\,\left( {r^{2} \,\frac{{\partial \sqrt {n_{e} } }}{\partial \,r}} \right)} \right]\, = \,0\,,$$

3 $$P_{e} \, = \,K_{e} \,n_{e}^{{\gamma_{e} }} \,.$$

The dynamics of the LNS^1,2,7,24,34^ can analogously be cast as

4 $$\frac{{\partial \,n_{l} }}{\partial \,t}\, + \,\frac{1}{{r^{2} }}\,\frac{\partial }{\partial r}\,\left( {r^{2} n_{l} u_{l} } \right)\, = \,0\,,$$

5 $$\left( {\frac{\partial }{\partial \,t}\, + \,u_{l} \frac{\partial }{\partial r}} \right)\,u_{l} \, + \,\left( {\frac{{Z_{l} e}}{{m_{l} }}} \right)\,\frac{\partial \Phi }{{\partial r}}\, + \,\frac{\partial \psi }{{\partial r}}\, + \,\frac{1}{{m_{l} n_{l} }}\,\frac{{\partial P_{l} }}{\partial r}\, = \,0\,,$$

6 $$P_{l} \, = \,n_{l} k_{B} T\, + \,B_{l} \,n_{l}^{2} \,.$$

Similarly, the dynamics of the HNS^1,2,7,24,34,35^ can be expressed as

7 $$\frac{{\partial \,n_{h} }}{\partial \,t}\, + \,\frac{1}{{r^{2} }}\,\frac{\partial }{\partial r}\,\left( {r^{2} n_{h} u_{h} } \right)\, = \,0\,,$$

8 $$\begin{aligned} & \left[ {1 + \tau_{m} \left( {\frac{\partial }{\partial t} + u_{h} \frac{\partial }{\partial r}} \right)} \right]\left[ {\left( {\frac{\partial }{\partial t} + u_{h} \frac{\partial }{\partial r}} \right)u_{h} + \left( {\frac{{eZ_{h} }}{{m_{h} }}} \right)\frac{\partial \Phi }{{\partial r}} + \frac{\partial \psi }{{\partial r}} + 2\Omega_{\varphi } v_{\theta } + \frac{1}{{m_{h} n_{h} }}\frac{{\partial P_{h} }}{\partial r}} \right] \\ & = \frac{1}{{m_{h} n_{h} }}\left( {\zeta + \frac{4}{3}\eta } \right)\frac{1}{{r^{2} }}\frac{\partial }{\partial r}\left( {r^{2} \frac{{\partial u_{h} }}{\partial r}} \right), \\ \end{aligned}$$

9 $$P_{h} \, = \,n_{h} k_{B} T + B_{h} \,n_{h}^{2} \,.$$

The Poisson equations describing the electrostatic and gravitational potential distributions originating from charged matter density fields are respectively given in generic notations^1,2,7^ as

10 $$\frac{1}{{r^{2} }}\frac{\partial }{\partial r}\left( {r^{2} \frac{\partial \Phi }{{\partial r}}} \right)\, = \,4\pi \,e\left( {n_{e} - Z_{l} n_{l} - Z_{h} n_{h} } \right)\,,$$

11 $$\frac{1}{{r^{2} }}\frac{\partial }{\partial r}\left( {r^{2} \frac{\partial \psi }{{\partial r}}} \right)\, = \,4\pi \,G\left( {m_{l} n_{l} + m_{h} n_{h} } \right)\,.$$

The notation $n_{s}$ stands for the population density associated with the *s*th species; *s* being *e* for the electrons, *l* for LNS, and *h* for HNS. $Z_{s} ,\,\,m_{s} ,\,\,P_{s} ,\,\,u_{s} \,$ signify the charge state, mass, pressure and flow speed of the *s*th species (*s* = *e, l, h*). *T* signifies the temperature of the system (in K). *B*_*l*_ and *B*_*h*_ are the electrostatic confinement constants associated with the LNS and HNS, respectively^24^. The azimuthal component of the angular velocity and polar component of the rotational velocity are respectively denoted as $\Omega_{\varphi }$ and $v_{\theta }$. $\Phi$ represents the electrostatic potential. $\psi$ is the gravitational potential. $k_{B} \, = \,1.38 \times 10^{ - 16} \,$ erg K^−1^ is the Boltzmann constant signifying the energy-temperature coupling. $G\, = \,6.67 \times 10^{ - 8}$ cm^3^ g^−1^ s^−2^ is the universal gravitational constant through which gravitating matter interacts. $\zeta$ and $\eta$ are the bulk viscosity (resistance to transverse flow) and shear viscosity (resistance to longitudinal flow) coefficients, respectively. $\tau_{m}$ is the viscoelastic relaxation time of the strongly coupled heavy nuclear fluid.

A number of noteworthy points regarding the above viscoelastic fluid picture are in order. The system is composed of strongly coupled HNS, weakly coupled LNS, and DES (both NR and UR)^1,2,7^. In simple terms, the Coulomb coupling parameter, that is $\Gamma$, is defined as the ratio of the mean potential energy per particle to the mean kinetic energy per particle^36^. For classical ions, $\Gamma = \,{{\left( {Ze^{2} } \right)} \mathord{\left/ {\vphantom {{\left( {Ze^{2} } \right)} {ak_{B} }}} \right. \kern-\nulldelimiterspace} {ak_{B} }}T$; where, $a \propto \,n^{{{{ - 1} \mathord{\left/ {\vphantom {{ - 1} 3}} \right. \kern-\nulldelimiterspace} 3}}}$ is the interparticle separation^7,37^. It is evident that $\Gamma > 1$ for the HNS due to their high charge and low temperature^36^. Thus, the HNS are strongly coupled^7^. The fact that kinetic energy of the HNS is comparatively low owing to their higher mass also adds to the reasons behind the HNS for being strongly coupled. Similarly, $\Gamma < 1$ for the LNS on account of their higher kinetic energy than the HNS. In other words, the LNS are weakly coupled. However, when the density becomes too high, i.e., when the interparticle separation becomes of the order of de-Broglie wavelength of electrons, classical treatment falls short. In these cases, we have the degeneracy parameter $\theta_{DP}$ which is defined as the ratio of the thermal energy to the Fermi energy^36^. For the electrons, $\theta_{DP} < < 1$ making the species degenerate where the quantum–mechanical effects play an important role^36^.

Equations (1), (4) and (7) are the equations of continuity for the DES, LNS, and HNS, respectively. It is seen from Eq. (2) that the forces arising due to the electrostatic pressure, degenerate quantum pressure, and quantum–mechanical Bohm potential pressure exactly balance each other to form a hydrostatic equilibrium system, at least initially. The inertial force has been ignored owing to the extremely small mass of the electrons. The effects of viscoelasticity become prominent only when the particles are strongly coupled (Coulomb coupling parameter exceeding unity)^37^, i.e., only for the HNS. Also, the Coriolis rotational force with all the usual notations^35^ is given as $F_{Co} = 2m\,\left| {\left( {\vec{v} \times \vec{\omega }} \right)} \right|$. Clearly, the rotational part, $\left| {\left( {\vec{v} \times \vec{\omega }} \right)} \right|$, is constant for a uniformly rotating plasma system. Thus, the effect of the Coriolis rotation becomes extremely small for the tiny electrons with negligible mass. It shows why the viscoelastic and rotational terms are not included in the momentum equation of the DES (Eq. 2). Due to similar reasons, the viscoelastic and rotational terms have been neglected in the momentum equation of the LNS (Eq. 5) as well. Equation (5) for the LNS is the analog of Eq. (2), where the forces due to their motion, electrostatic potential, gravitational potential, and pressure exactly balance each other. The force-balancing condition for the HNS is given by Eq. (8) ^1,2,7,35^, where the various forces on the HNS exerted by virtue of their inertia, electrostatic potential, gravitational potential, composite pressure, and Coriolis rotation are exactly balanced by the dissipative viscoelastic forces. The main reason for the difference in the momentum equations of the classical LNS (Eq. 5) and HNS (Eq. 8) lies in the difference of their coupling parameter regimes (weak LNS and strong HNS). It is to be noted that when $\tau_{m} = 0$, Eq. (8) reduces to the Navier–Stokes hydrodynamic equation. In the limit $\tau_{m} \to \infty$, the species shows solid-like behaviour. Thus, our region of interest here is the viscoelastic fluid region existing in the parametric window defined by $0 < \tau_{m} < \infty$ regime^37^. Rapidly rotating collapsing white dwarfs are highly viscous in nature^30^. Thus, the effects of the Coriolis rotation and viscoelasticity become relevant for a rapidly rotating contracting white dwarf in the stage of shedding its mass^30^. Also, both the viscoelasticity and rotation play an important role in the evolution of white dwarf merger remnants^31^.

Besides, the equations of macroscopic state describing the constitutive species are respectively represented by Eqs. (3), (6) and (9). Both Eqs. (3) and (6) give the effective pressure acting on the DES and the LNS in our model set up, respectively. Equation (6) gives the effective pressure acting on the LNS, i.e., the sum of the thermal pressure and ECP. In contrast, in dwarf plasmas, the degenerate pressure of the electrons far exceeds all other pressures acting on the species, such as the electron thermal pressure, ECP, etc. It hereby makes the degenerate electron pressure significantly prevail only on the quantum DES. Thus, Eq. (3) gives the non-thermal degenerate pressure operating most significantly on the DES in our dwarf plasma system. Similarly, Eq. (9) is the classical analog of Eq. (3), but for the HNS. The electro-gravitational Poisson equations, as given respectively by Eqs. (10) and (11), may look to be time-stationary in nature because of the conservative nature of the long-range electro-gravitational force fields. Clearly, the self-interactions of matter relative to the electric and gravitational fields remain always invariant in the classical NR regime.

In order for a scale-invariant stability analysis of the proposed model, we apply a standard scheme of astronomical normalization^1,2,7,24^. As a result, the scale-invariant dimensionless set of the basic governing equations (Eqs. 1 and 11) describing our model read respectively in the customary notations^1,2,7,24^ as

12 $$\frac{{\partial N_{e} }}{\partial \tau } + \frac{1}{{\xi^{2} }}\frac{\partial }{\partial \xi }\left( {\xi^{2} N_{e} M_{e} } \right) = 0\,\,,$$

13 $$N_{e} \frac{{\partial \Phi_{E} }}{\partial \xi } - K^{\prime}_{e} \gamma_{e} N_{e}^{{\gamma_{e\,} - 1}} \frac{{\partial N_{e} }}{\partial \xi } - \frac{1}{2}H^{{\prime}{2}} M_{Fe}^{2} \left[ {\frac{1}{2}\frac{{\partial^{3} N_{e} }}{{\partial \xi^{3} }} + \frac{1}{\xi }\frac{{\partial^{2} N_{e} }}{{\partial \xi^{2} }} - \frac{1}{{\xi^{2} }}\frac{{\partial N_{e} }}{\partial \xi }} \right]\, = \,0\,,$$

14 $$\frac{{\partial N_{l} }}{\partial \tau } + \frac{1}{{\xi^{2} }}\frac{\partial }{\partial \xi }\left( {\xi^{2} N_{l} M_{l} } \right) = 0\,\,\,,$$

15 $$\left( {\frac{\partial }{\partial \tau } + M_{l} \frac{\partial }{\partial \xi }} \right)M_{l} N_{l} + N_{l} \frac{{\partial \Phi_{E} }}{\partial \xi } + N_{l} \frac{\partial \Psi }{{\partial \xi }} + A^{\prime}_{l} \frac{\partial }{\partial \xi }\left[ {N_{l} T^{*} + B_{l}^{*} N_{l}^{2} } \right] = 0\,,$$

16 $$\frac{{\partial N_{h} }}{\partial \tau } + \frac{1}{{\xi^{2} }}\frac{\partial }{\partial \xi }\left( {\xi^{2} N_{h} M_{h} } \right) = 0\,\,,$$

17 $$\begin{aligned} & \left[ {1 + \tau_{m}^{*} \frac{\partial }{\partial \tau }} \right]\left[ {\left( {\frac{\partial }{\partial \tau } + M_{h} \frac{\partial }{\partial \xi }} \right)M_{h} N_{h} + \beta N_{h} \frac{{\partial \Phi_{E} }}{\partial \xi } + N_{h} \frac{\partial \Psi }{{\partial \xi }} + 2N_{h} C_{F}^{*} + A^{\prime}_{h} \frac{\partial }{\partial \xi }\left( {N_{h} T^{*} + B_{h}^{*} N_{h}^{2} } \right)} \right] \\ & = \chi^{*} \frac{1}{{\xi^{2} }}\frac{\partial }{\partial \xi }\left( {\xi^{2} \frac{{\partial M_{h} }}{\partial \xi }} \right), \\ \end{aligned}$$

18 $$\frac{1}{{\xi^{2} }}\frac{\partial }{\partial \xi }\left( {\xi^{2} \frac{{\partial \Phi_{E} }}{\partial \xi }} \right) = \left( {1 + \mu } \right)N_{e} - N_{l} - \mu N_{h} \,,$$

19 $$\frac{1}{{\xi^{2} }}\frac{\partial }{\partial \xi }\left( {\xi^{2} \frac{\partial \Psi }{{\partial \xi }}} \right) = \sigma \,\left( {N_{l} + \frac{\mu }{\beta }N_{h} } \right)\,,$$

where, $\xi = \,{r \mathord{\left/ {\vphantom {r {\lambda_{Dl} }}} \right. \kern-\nulldelimiterspace} {\lambda_{Dl} }}$ is the normalized radial coordinate with the normalization parameter given as $\lambda_{Dl} = \left( {{{m_{e} c^{2} } \mathord{\left/ {\vphantom {{m_{e} c^{2} } {4\,\pi \,n_{l0} Z_{l} e^{2} }}} \right. \kern-\nulldelimiterspace} {4\,\pi \,n_{l0} Z_{l} e^{2} }}} \right)^{{{1 \mathord{\left/ {\vphantom {1 2}} \right. \kern-\nulldelimiterspace} 2}}}$. $\tau = \,\,{t \mathord{\left/ {\vphantom {t {\omega_{pl}^{ - 1} }}} \right. \kern-\nulldelimiterspace} {\omega_{pl}^{ - 1} }}$ is the normalized time coordinate. $\tau_{m}^{*} = {{\tau_{m} } \mathord{\left/ {\vphantom {{\tau_{m} } {\omega_{pl}^{ - 1} }}} \right. \kern-\nulldelimiterspace} {\omega_{pl}^{ - 1} }}$ is the normalized viscoelastic relaxation time. The time normalization factor is the light nuclear plasma oscillation time scale given as: $t_{pl} = \omega_{pl}^{ - 1} = \left( {{{m_{l} } \mathord{\left/ {\vphantom {{m_{l} } {4\,\pi \,n_{l0} Z_{l}^{2} e^{2} }}} \right. \kern-\nulldelimiterspace} {4\,\pi \,n_{l0} Z_{l}^{2} e^{2} }}} \right)^{{{1 \mathord{\left/ {\vphantom {1 2}} \right. \kern-\nulldelimiterspace} 2}}}$. $Z^{\prime} = {{Z_{h} } \mathord{\left/ {\vphantom {{Z_{h} } {Z_{l} }}} \right. \kern-\nulldelimiterspace} {Z_{l} }}$ denotes the ratio of the heavy-to-light nuclear charge number. $\mu = \,{{Z^{\prime}n_{h0} } \mathord{\left/ {\vphantom {{Z^{\prime}n_{h0} } {n_{l0} }}} \right. \kern-\nulldelimiterspace} {n_{l0} }}$ stands for the ratio of the charge densities of the heavy-to-light nuclear species. The relative nuclear charge-to-mass coupling parameter is denoted by $\beta = Z^{\prime}{{m_{l} } \mathord{\left/ {\vphantom {{m_{l} } {m_{h} }}} \right. \kern-\nulldelimiterspace} {m_{h} }}$. The population densities of the constitutive particles have been normalized by their equilibrium number density as $N_{s} = {{n_{s} } \mathord{\left/ {\vphantom {{n_{s} } {n_{s0} }}} \right. \kern-\nulldelimiterspace} {n_{s0} }}$. The squared Fermi Mach number is given by $M_{Fe}^{2} = {{v_{Fe}^{4} } \mathord{\left/ {\vphantom {{v_{Fe}^{4} } {C_{l}^{2} }}} \right. \kern-\nulldelimiterspace} {C_{l}^{2} }}c^{2}$. Likewise, the normalized form of the fluid flow velocity is given by $M_{s} = {{u_{s} } \mathord{\left/ {\vphantom {{u_{s} } {C_{l} }}} \right. \kern-\nulldelimiterspace} {C_{l} }}$, where $C_{l} = \left( {{{Z_{l} \,m_{e} c^{2} } \mathord{\left/ {\vphantom {{Z_{l} \,m_{e} c^{2} } {m_{l} }}} \right. \kern-\nulldelimiterspace} {m_{l} }}} \right)^{{{1 \mathord{\left/ {\vphantom {1 2}} \right. \kern-\nulldelimiterspace} 2}}}$ gives the rescaled light nuclear transit speed. $H^{\prime} = {{\hbar \omega_{pl}^{{}} } \mathord{\left/ {\vphantom {{\hbar \omega_{pl}^{{}} } {m_{e}^{{}} }}} \right. \kern-\nulldelimiterspace} {m_{e}^{{}} }}v_{Fe}^{2}$ denotes the quantum parameter signifying the ratio between the plasmon energy associated with the light nucleus and the Fermi energy associated with degenerate electrons. The ratio between the square of the Jeans frequency to that of the light nuclear plasma oscillation frequency is given as $\sigma = {{\omega_{Jl}^{2} } \mathord{\left/ {\vphantom {{\omega_{Jl}^{2} } {\omega_{pl}^{2} }}} \right. \kern-\nulldelimiterspace} {\omega_{pl}^{2} }}$, where $\omega_{Jl} = \sqrt {4\,\pi \,Gn_{l0} m_{l} }$. $A^{\prime}_{l} \, = \,{{m_{e} c^{2} } \mathord{\left/ {\vphantom {{m_{e} c^{2} } {m_{l} }}} \right. \kern-\nulldelimiterspace} {m_{l} }}C_{l}^{2}$ stands for the ratio of the relativistic electronic energy to the LNS energy. $A^{\prime}_{h} = \,{{m_{e} c^{2} } \mathord{\left/ {\vphantom {{m_{e} c^{2} } {m_{h} C_{l}^{2} }}} \right. \kern-\nulldelimiterspace} {m_{h} C_{l}^{2} }}$ is the analogous term for the HNS. The constants $B_{l}^{*}$ and $B_{h}^{*}$ have been normalized as $B_{l}^{*} = {{B_{l} n_{l0} } \mathord{\left/ {\vphantom {{B_{l} n_{l0} } {m_{e} c^{2} }}} \right. \kern-\nulldelimiterspace} {m_{e} c^{2} }}$ and $B_{h}^{*} = \,\,{{B_{h} n_{h0} } \mathord{\left/ {\vphantom {{B_{h} n_{h0} } {m_{e} }}} \right. \kern-\nulldelimiterspace} {m_{e} }}c^{2}$, respectively. $T^{*} = {{k_{B} T} \mathord{\left/ {\vphantom {{k_{B} T} {m_{e} }}} \right. \kern-\nulldelimiterspace} {m_{e} }}c^{2}$ stands for the normalized isothermal nuclear plasma temperature of the bulk plasma fluid. The effective generalized viscosity given by $\chi = \left( {\zeta + {{4\eta } \mathord{\left/ {\vphantom {{4\eta } 3}} \right. \kern-\nulldelimiterspace} 3}} \right)$ has been normalized as $\chi^{*} = {\chi \mathord{\left/ {\vphantom {\chi {m_{h} }}} \right. \kern-\nulldelimiterspace} {m_{h} }}n_{h0} C_{l} \lambda_{Dl}$. The polytropic constant for the electronic dynamics in the normalized form is given as $K^{\prime}_{e} = {{K_{e} \,n_{e0}^{{\gamma_{e} - 1}} } \mathord{\left/ {\vphantom {{K_{e} \,n_{e0}^{{\gamma_{e} - 1}} } {m_{e} c^{2} }}} \right. \kern-\nulldelimiterspace} {m_{e} c^{2} }}$. The normalized Coriolis force is denoted as $C_{F}^{*} = \Omega_{\varphi }^{*} M_{h\theta }$, where the azimuthal component of angular velocity and polar component of the rotational velocity are normalized as $\Omega_{\varphi }^{*} = {{\Omega_{\varphi } } \mathord{\left/ {\vphantom {{\Omega_{\varphi } } {\omega_{pl} }}} \right. \kern-\nulldelimiterspace} {\omega_{pl} }}$ and $M_{h\theta } = {{v_{\theta } } \mathord{\left/ {\vphantom {{v_{\theta } } {C_{l} }}} \right. \kern-\nulldelimiterspace} {C_{l} }}$, respectively. $\Phi_{E} = e{{\Phi \,} \mathord{\left/ {\vphantom {{\Phi \,} {m_{e} c^{2} }}} \right. \kern-\nulldelimiterspace} {m_{e} c^{2} }}$ gives the normalized electrostatic potential arising due to local plasma polarization effects. The normalized gravitational potential is given as ${{\Psi = \psi } \mathord{\left/ {\vphantom {{\Psi = \psi } {C_{l}^{2} }}} \right. \kern-\nulldelimiterspace} {C_{l}^{2} }}$.

Three appendices are concisely added at the last to depict the entire scheme of abbreviations (ESM Appendix-A), symbolic normalization (ESM Appendix-B), and point-wise difference between ion-acoustic waves (IAWs) and the NAWs (ESM Appendix-C) for the sake of instant reference of the readers of this contribution.

### Perturbation analysis

We linearly perturb the relevant physical fluid parameters appearing in Eqs. (12)–(19), which govern the complex system dynamics under consideration, using a standard spherical wave analysis^1^ in a self-consistently auto-normalized Fourier form as

20 $$F\left( {\xi ,\,\tau } \right) = F_{0} + \frac{1}{\xi }F_{1} \exp \left[ { - i\left( {\Omega \tau - k^{*} \xi } \right)} \right]\,\,,$$

21 $$F = \left[ {N_{j} \,\,\,\,\,\,\,\,M_{j} \,\,\,\,\,\,\,\,\Phi_{E} \,\,\,\,\,\,\,\,\Psi } \right]^{T} ,$$

22 $$F_{0} = \left[ {1\,\,\,\,\,\,\,\,\,\,\,\,\,0\,\,\,\,\,\,\,\,\,\,\,\,\,\,0\,\,\,\,\,\,\,\,\,\,\,\,\,\,0} \right]^{T} \,,$$

23 $$F_{1} = \left[ {N_{j1} \,\,\,\,M_{j1} \,\,\,\,\,\,\Phi_{E1} \,\,\,\,\,\,\Psi_{1} } \right]^{T} .$$

﻿Here, *F*_*1*_ denotes the perturbations evolving radially about their corresponding hydrostatic homogeneous equilibrium values *F*_*0*_. The perturbed set of equations, after linearization relative to the defined equilibrium, are given in ESM Appendix-D. Application of Eq. (20) results in a Fourier wave space $\left( {\,\Omega \,,\,\,k^{*} } \right)$; where, the linear spatio-temporal operators transform as ${\partial \mathord{\left/ {\vphantom {\partial {\partial \xi \to \left( {\,ik^{*} - \,\,{1 \mathord{\left/ {\vphantom {1 \xi }} \right. \kern-\nulldelimiterspace} \xi }} \right)}}} \right. \kern-\nulldelimiterspace} {\partial \xi \to \left( {\,ik^{*} - \,\,{1 \mathord{\left/ {\vphantom {1 \xi }} \right. \kern-\nulldelimiterspace} \xi }} \right)}}$ and ${\partial \mathord{\left/ {\vphantom {\partial {\partial \tau \to \left( { - i\,\,\Omega } \right)}}} \right. \kern-\nulldelimiterspace} {\partial \tau \to \left( { - i\,\,\Omega } \right)}}$, respectively. In the new wave space $\left( {\,\Omega \,,\,\,k^{*} } \right)$, the linearly perturbed parametric quantities from Eqs. (12) to (19) can respectively be given in an algebraic form as

24 $$N_{e1} = \frac{1}{i\Omega }\left( {ik^{*} + \frac{1}{\xi }} \right)M_{e1} \,\,,$$

25 $$M_{e1} = i\Omega \,\Phi_{E1} \left( {ik^{*} - \frac{1}{\xi }} \right)\,\left[ {\left( {k^{{*^{2} }} + \frac{1}{{\xi^{2} }}} \right)\left( {\frac{1}{4}H^{{\prime}{2}} M_{Fe}^{2} k^{{*^{2} }} - K^{\prime}\gamma_{e} } \right)} \right]^{ - 1} ,$$

26 $$N_{l1} = \frac{1}{i\Omega }\left( {ik^{*} + \frac{1}{\xi }} \right)M_{l1} \,\,,$$

27 $$M_{l1} = - \frac{\sigma \mu }{\beta }\frac{1}{{k^{{*^{2} }} }}\left( {k^{{*^{2} }} + \frac{1}{{\xi^{2} }}} \right)\frac{{M_{h1} }}{{\left( {\Omega^{2} + E} \right)}} - i\Omega \left( {ik^{*} - \frac{1}{\xi }} \right)\frac{{\Phi_{E1} }}{{\left( {\Omega^{2} + E} \right)}}\,\,\,\,,$$

28 $$N_{h1} = \frac{1}{i\Omega }\left( {ik^{*} + \frac{1}{\xi }} \right)M_{h1} \,\,,$$

29 $$M_{h1} = \Phi_{E1} \left( { - ik^{*} + \frac{1}{\xi }} \right)\left[ { - \frac{\sigma }{{k^{{*^{2} }} }}\left( {k^{{*^{2} }} + \frac{1}{{\xi^{2} }}} \right)\frac{1}{{\left( {\Omega^{2} + E} \right)}} + \beta } \right]\,\left[ { - i\Omega + \frac{1}{i\Omega }H + \frac{{\chi^{*} k^{{*^{2} }} }}{{\left( {1 - i\Omega \tau_{m}^{*} } \right)}}} \right]^{ - 1} ,$$

30 $$\Phi_{E1} = - \left[ {\left( {1 + \mu } \right)N_{e1} - \mu N_{h1} - N_{l1} } \right]\frac{1}{{k^{{*^{2} }} }}\,\,,$$

31 $$\Psi_{1} = - \left( {N_{l1} + \frac{\mu }{\beta }N_{h1} } \right)\frac{\sigma }{{k^{{*^{2} }} }}\,\,.$$

where,

32 $$E = \left( {k^{{*^{2} }} + \frac{1}{{\xi^{2} }}} \right)\,\left( {\frac{\sigma }{{k^{{*^{2} }} }} - A^{\prime}_{l} \,\left( {T^{*} + 2B_{l}^{*} } \right)} \right)\,;$$

33 $$H = \left[ {2C_{F}^{*} \left( {ik^{*} + \frac{1}{\xi }} \right) - A^{\prime}_{h} \,\left( {T^{*} + 2B_{h}^{*} } \right)\left( {k^{{*^{2} }} + \frac{1}{{\xi^{2} }}} \right) + \frac{\sigma \mu }{\beta }\frac{1}{{k^{{*^{2} }} }}\left( {k^{{*^{2} }} + \frac{1}{{\xi^{2} }}} \right)\left\{ { - \frac{\sigma }{{k^{{*^{2} }} }}\left( {k^{{*^{2} }} + \frac{1}{{\xi^{2} }}} \right)\frac{1}{{\left( {\Omega^{2} + E} \right)}} + 1} \right\}} \right]\,\,.$$

Applying a standardized analytical method of substitution, elimination, and decomposition in the linearized set of equations (ESM Appendix-D), we obtain a generalized linear dispersion relation (septic) of a unique shape given as

34 $$A_{7} \Omega^{7} \, + A_{6} \Omega^{6} + A_{5} \Omega^{5} + A_{4} \Omega^{4} + A_{3} \Omega^{3} + A_{2} \Omega^{2} + A_{1} \Omega + A_{0} = 0;$$

where, the different atypical coefficients in an extended form are respectively given as

35 $$A_{7} = \left[ { - i\tau_{m}^{*} \left( { - k^{{*^{2} }} + F} \right)} \right],$$

36 $$A_{6} = \left( { - k^{{*^{2} }} + F} \right),$$

37 $$A_{5} = \left[ { - i\tau_{m}^{*} \left\{ {\left( {k^{{*^{2} }} + \frac{1}{{\xi^{2} }}} \right)\,\left( {1 + \mu \beta } \right) + \left( { - k^{{*^{2} }} + F} \right)\,\left( {2E + Q - \frac{{k^{{*^{2} }} \chi^{*} }}{{\tau_{m}^{*} }}} \right)} \right\}} \right],$$

38 $$A_{4} = \left[ {\left( {k^{{*^{2} }} + \frac{1}{{\xi^{2} }}} \right)\,\left( {1 + \mu \beta } \right) + \left( { - k^{{*^{2} }} + F} \right)\,\left( {2E + Q} \right)} \right],$$

39 $$\begin{aligned} A_{3} & = \left[ { - i\tau_{m}^{*} \left\{ {\mu \left( {k^{{*^{2} }} + \frac{1}{{\xi^{2} }}} \right)\,\left( {2E\beta - \frac{2\sigma }{{k^{{*^{2} }} }}\left( {k^{{*^{2} }} + \frac{1}{{\xi^{2} }}} \right) + \frac{E}{\mu }} \right) + \left\{ {2E\left( { - k^{{*^{2} }} + F} \right) + \left( {k^{{*^{2} }} + \frac{1}{{\xi^{2} }}} \right)} \right\}\left( {Q - \frac{{k^{{*^{2} }} \chi^{*} }}{{\tau_{m}^{*} }}} \right)} \right\}} \right] \\ & \quad + \left[ { - i\tau_{m}^{*} \left( { - k^{{*^{2} }} + F} \right)\left\{ {E^{2} - \frac{\mu }{\beta }\left( {\frac{\sigma }{{k^{{*^{2} }} }}\left( {k^{{*^{2} }} + \frac{1}{{\xi^{2} }}} \right)} \right)^{2} } \right\}} \right], \\ \end{aligned}$$

40 $$\begin{aligned} A_{2} & = \left[ {\mu \left( {k^{{*^{2} }} + \frac{1}{{\xi^{2} }}} \right)\left( {2E\beta - \frac{2\sigma }{{k^{{*^{2} }} }}\left( {k^{{*^{2} }} + \frac{1}{{\xi^{2} }}} \right) + \frac{E}{\mu }} \right) + \left\{ {2E\left( { - k^{{*^{2} }} + F} \right) + \left( {k^{{*^{2} }} + \frac{1}{{\xi^{2} }}} \right)} \right\}Q} \right] \\ & \quad + \left[ {\left( { - k^{{*^{2} }} + F} \right)\left\{ {E^{2} - \frac{\mu }{\beta }\left( {\frac{\sigma }{{k^{{*^{2} }} }}\left( {k^{{*^{2} }} + \frac{1}{{\xi^{2} }}} \right)} \right)^{2} } \right\}} \right], \\ \end{aligned}$$

41 $$\begin{aligned} A_{1} & = \left[ { - i\tau_{m}^{*} \left\{ {\frac{\mu }{\beta }\left( {k^{{*^{2} }} + \frac{1}{{\xi^{2} }}} \right)\left\{ {\beta^{2} E^{2} - \frac{\sigma }{{k^{{*^{2} }} }}\left( {k^{{*^{2} }} + \frac{1}{{\xi^{2} }}} \right)\,\left( {2\beta \,E + \frac{\sigma }{{k^{{*^{2} }} }}E\left( { - k^{{*^{2} }} + F} \right)} \right)} \right\}} \right\}} \right] \\ & \quad + \left[ { - i\tau_{m}^{*} \left\{ {E^{2} \left( { - k^{{*^{2} }} + F} \right) + E\left( {k^{{*^{2} }} + \frac{1}{{\xi^{2} }}} \right)} \right\}\left( {Q - \frac{{k^{{*^{2} }} \chi^{*} }}{{\tau_{m}^{*} }}} \right)} \right], \\ \end{aligned}$$

42 $$\begin{aligned} A_{0} & = \left[ {\frac{\mu }{\beta }\left( {k^{{*^{2} }} + \frac{1}{{\xi^{2} }}} \right)\left\{ {\beta^{2} E^{2} - \frac{\sigma }{{k^{{*^{2} }} }}\left( {k^{{*^{2} }} + \frac{1}{{\xi^{2} }}} \right)\left( {2\beta \,E + \frac{\sigma }{{k^{{*^{2} }} }}E\left( { - k^{{*^{2} }} + F} \right)} \right)} \right\}} \right] \\ & \quad + \left[ {\left\{ {E^{2} \left( { - k^{{*^{2} }} + F} \right) + E\left( {k^{{*^{2} }} + \frac{1}{{\xi^{2} }}} \right)} \right\}Q} \right]. \\ \end{aligned}$$

In the ultra-low frequency limit ($\Omega^{a} = 0\,\forall \,a\, > 1$), Eq. (34) gets reduced to

43 $$\Omega \,\, = iP\,\,\left[ {\left\{ {\left( {E^{2} \left( { - k^{{*^{2} }} + F} \right) + E\left( {k^{{*^{2} }} + \frac{1}{{\xi^{2} }}} \right)} \right) \times k^{{*^{2} }} \chi^{*} } \right\} - \tau_{m}^{*} P} \right]^{ - 1} .$$

The various new terms substituted in Eqs. (35)–(43) in generic notations are given as

44 $$F = \left( {1 + \mu } \right)\,\,\left( {\frac{1}{4}H^{{\prime}{2}} M_{Fe}^{2} k^{{*^{2} }} - K^{\prime}\gamma_{e} } \right)^{ - 1} \,\,,$$

45 $$Q = \left[ {2C_{F}^{*} \left( {ik^{*} + \frac{1}{\xi }} \right) + \left( {k^{{*^{2} }} + \frac{1}{{\xi^{2} }}} \right)\,\left( {\frac{\sigma \mu }{\beta }\frac{1}{{k^{{*^{2} }} }} - A^{\prime}_{h} \,\left( {T^{*} + 2B_{h}^{*} } \right)} \right)\,} \right]\,\,,$$

46 $$P = \left[ {\frac{\mu }{\beta }\,\,\left( {k^{{*^{2} }} + \frac{1}{{\xi^{2} }}} \right)\,\,\left\{ {\beta^{2} E - \,\frac{\sigma }{{k^{{*^{2} }} }}\left( {k^{{*^{2} }} + \frac{1}{{\xi^{2} }}} \right)\,\,\left( {2\beta \, + \frac{\sigma }{{k^{{*^{2} }} }}\left( { - k^{{*^{2} }} + F} \right)} \right)} \right\}\, + \,\left\{ {E\,\left( { - k^{{*^{2} }} + F} \right) + \left( {k^{{*^{2} }} + \frac{1}{{\xi^{2} }}} \right)\,} \right\}Q} \right]\,\,.$$

It is now quite evident that the proposed dispersion analysis has multiparametric dependencies through the coefficients (Eqs. 35–42) on the proposed plasma model configuration, intercoupled via the explicit parametric functional forms (Eqs. 44–46).

## Results and discussions

**Figure 1:**
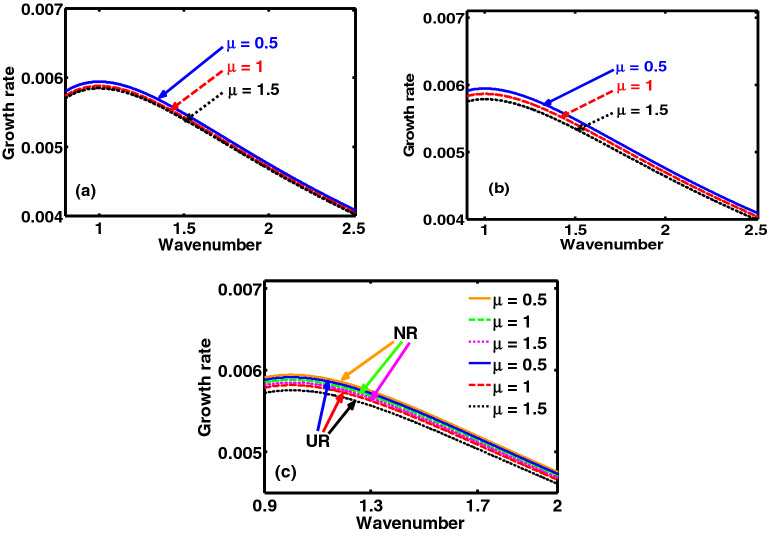
Profile of the normalized growth rate ($\Omega_{i}$) with variation in the normalized angular wavenumber ($k^{*}$) for different values of the charge density ratio of the heavy-to-light nuclear species ($\mu = {{Z_{h} n_{h0} } \mathord{\left/ {\vphantom {{Z_{h} n_{h0} } {Z_{l} n_{l0} }}} \right. \kern-\nulldelimiterspace} {Z_{l} n_{l0} }}$). The different subplots link to the (**a**) NR limit, (**b**) UR limit and (**c**) NR and UR limits conjointly, respectively.

**Figure 2:**
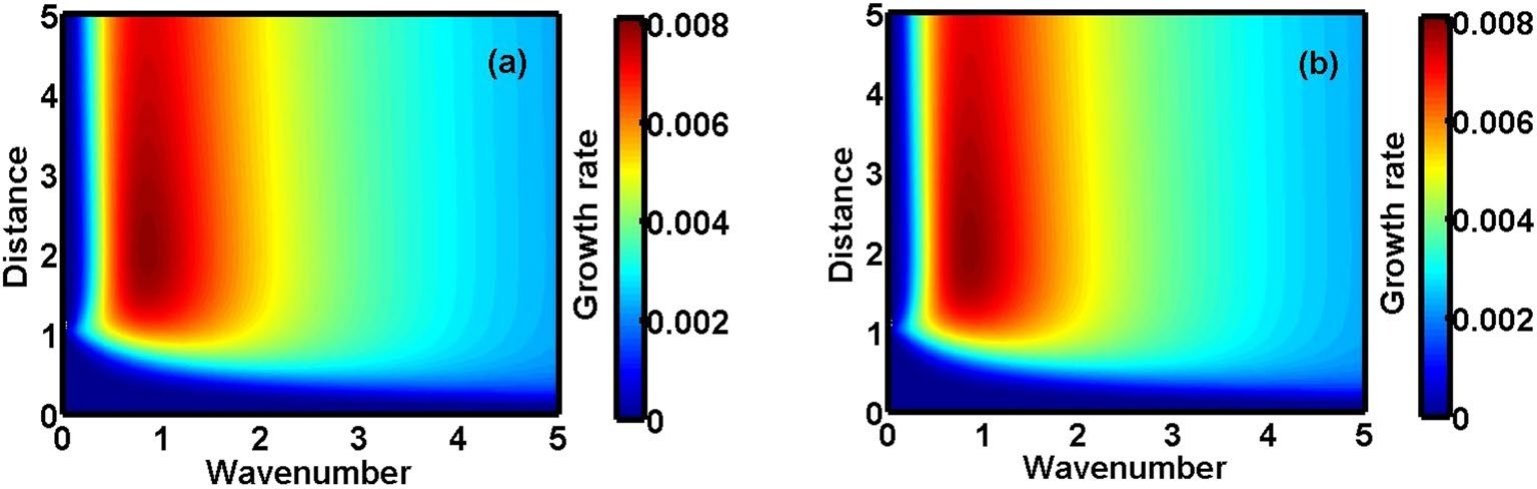
Colourspectral profile for the normalized growth rate for $\mu \, = \,0.5$ in the (**a**) NR and (**b**) UR limits, respectively.

**Figure 3:**
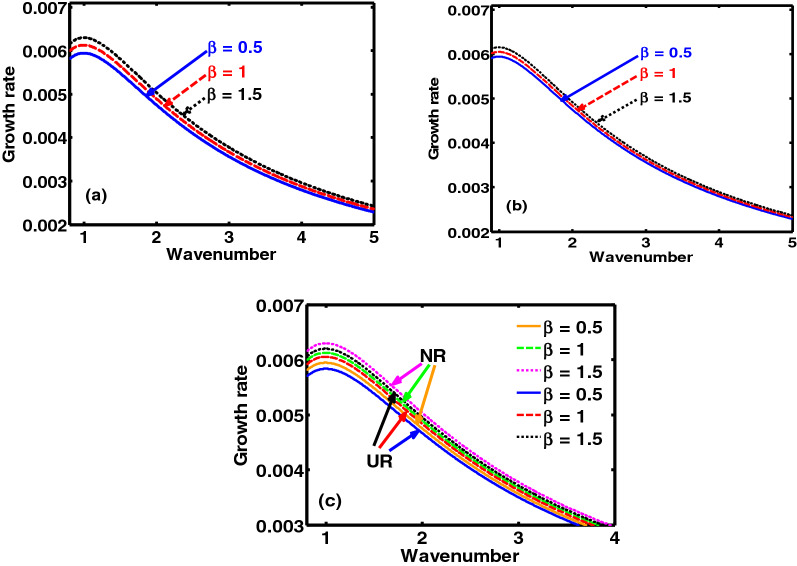
Same as Fig. 1, but for different values of charge-to-mass coupling parameter ($\beta = {{Z_{h} m_{l} } \mathord{\left/ {\vphantom {{Z_{h} m_{l} } {Z_{l} }}} \right. \kern-\nulldelimiterspace} {Z_{l} }}m_{h}$) in the (**a**) NR limit, (**b**) UR limit, (**c**) NR and UR limits, respectively.

**Figure 4:**
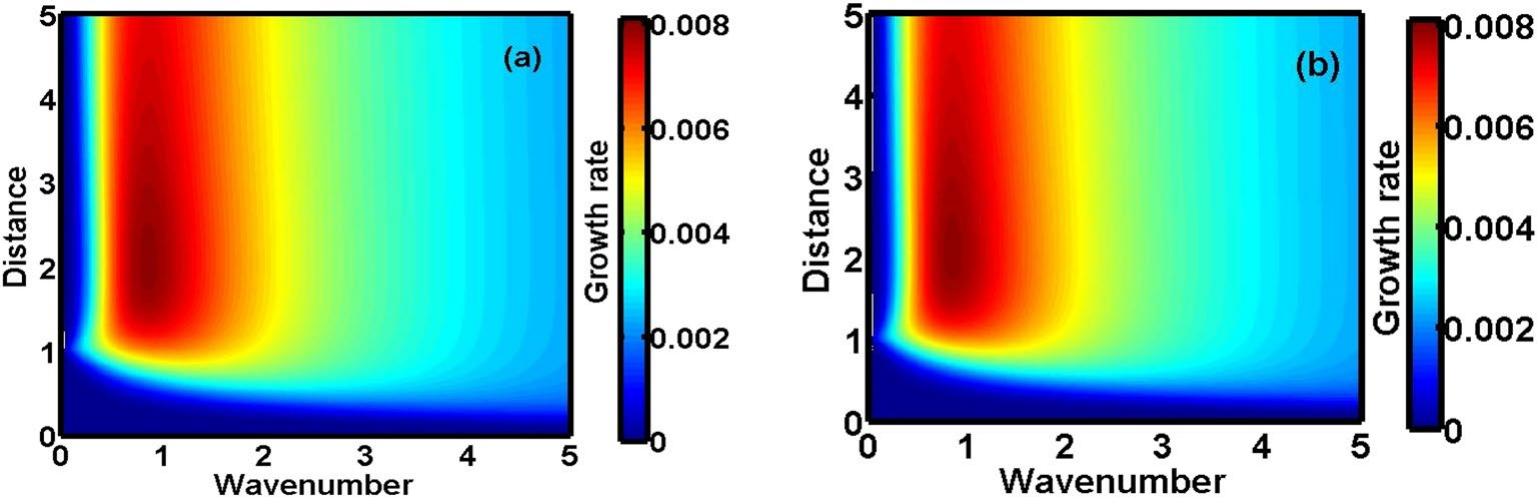
Same as Fig. 2, but for $\beta = 0.5$ in the (**a**) NR limit and (**b**) UR limit, respectively.

**Figure 5:**
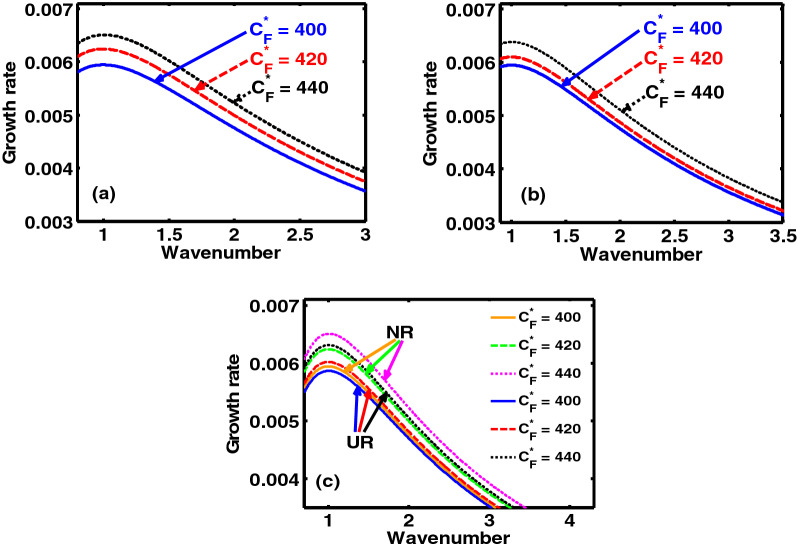
Same as Fig. 1, but for different values of the Coriolis force **(**$C_{F}^{*}$**)** in the (**a**) NR limit, (**b**) UR limit, (**c**) NR and UR limit, respectively.

**Figure 6:**
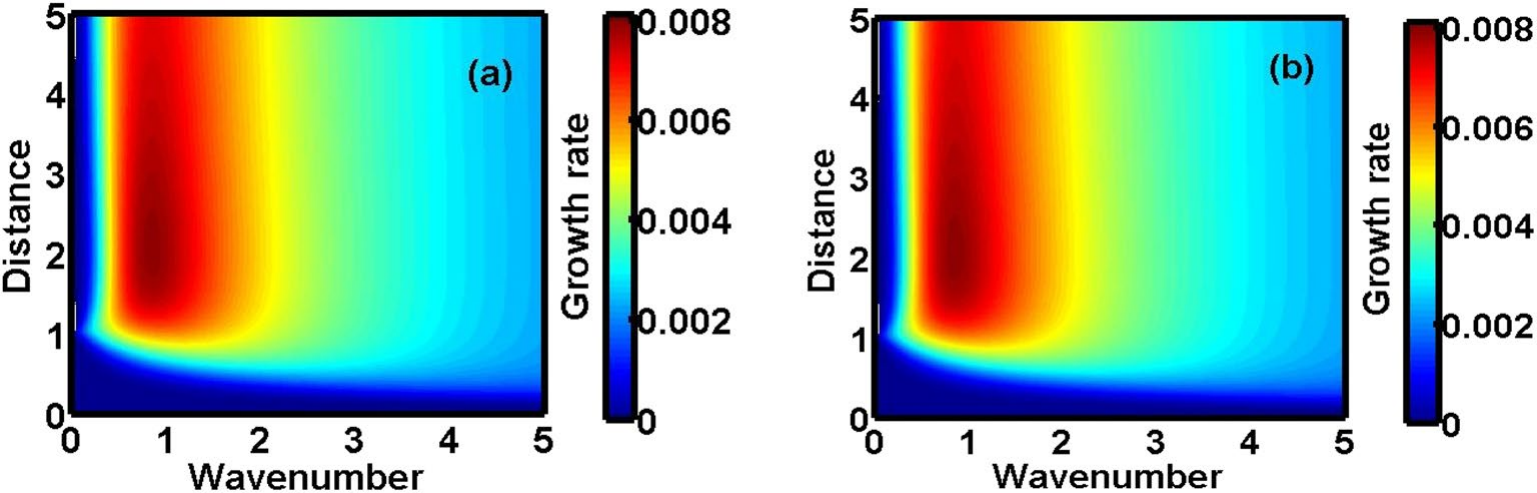
Same as Fig. 2, but for $C_{F}^{*} = 400$ in the (**a**) NR limit and (**b**) UR limit, respectively.

The semi-analytic study proposed here puts forward a theoretic model to investigate the excitation and propagatory dynamics of the NAWs in a rotating, self-gravitating, electrostatically confined DQP system. The considered model is set up in the light of a spherically symmetric geometric construct. The concurrent influence of the Bohm potential, ECP, Coriolis rotation, self-gravity, and viscoelasticity is appropriately included. A linear normal mode analysis over the perturbed DQP system yields a generalized dispersion relation (septic) of a unique pattern, characterizing the NAWs excitable in the system. A numerical illustrative platform is provided to reveal the microphysical dynamics of the derived dispersion law, which is, in fact, validated in the ultra-low frequency approximation. The growth rates of the model system fluctuations with variation in the normalized wavenumber, with minor differences for both the NR, and UR limits, are illustrated pictorially in Figs. 1, 2, 3, 4, 5 and 6.

In Fig. 1, we depict the profile structures of the growth rate with variation in the wavenumber for different values of the charge density ratio of the heavy-to-light nuclear species ($\mu = {{Z_{h} n_{h0} } \mathord{\left/ {\vphantom {{Z_{h} n_{h0} } {Z_{l} }}} \right. \kern-\nulldelimiterspace} {Z_{l} }}n_{l0}$). The different subplots link to the (a) pure NR limit, (b) pure UR limit, (c) conjoint NR-UR limit, respectively. The different multiparametric input values used here are taken from the literature^1,2,7,24,28,34^ given as: $\sigma = 10^{ - 2}$, $\xi = 1$, $A^{\prime}_{l} = 10$, $A^{\prime}_{h} = 10^{2}$, $\tau_{m}^{*} = 10^{ - 2}$, $H^{\prime} = 0.1$, $M_{Fe} = 1$, $\chi^{*} = 10^{ - 3}$, $C_{F}^{*} = 400$, $B_{l}^{*} = 4$, $B_{h}^{*} = 4$, $\beta = 1$. As clearly visible from the distinct coloured lines (Fig. 1), $\mu$ acts as a stabilizing agency for the fluctuations. It can be physically attributed to the dominancy of the inertial force imposed by the HNS over the elasticity contributed jointly by the LNS and DES. Our model mimics the environ of a rapidly rotating contracting white dwarf star. If the contraction is large enough to increase the number density of heavy nuclei (number of nuclei present per unit volume), the value of $\mu$ gradually increases. The heavier nuclei are larger in size as compared to the lighter nuclei. It is because the nuclear size goes as, $R = f\left( A \right) = R_{0} A^{{{1 \mathord{\left/ {\vphantom {1 3}} \right. \kern-\nulldelimiterspace} 3}}}$, where $R_{0} = 1.2 \times 10^{ - 17}$ cm and *A* is the mass number of the nucleus. It is to be noted that the contraction in the dwarf plasma volume results in an increase in the number density of the lighter nuclei. It, however, results in more closeness of heavy nuclei (bigger) than that of lighter nuclei (smaller). It increases the inertial action of the HNS, thereby suppressing the instability growth rate. Thus, $\mu$ introduces a stabilizing influence on the growth. For a better confirmatory visualization on a colour phase space ($k^{*} ,\,\xi$), Fig. 2 shows the colour spectral profiles of the growth rate with variation in the radial distance and wavenumber for $\mu = 0.5$ in the (a) NR limit and (b) UR limit.

As in Fig. 3, we show the same as Fig. 1, but for different values of the charge-to-mass coupling parameter ($\beta = {{Z_{h} m_{l} } \mathord{\left/ {\vphantom {{Z_{h} m_{l} } {Z_{l} }}} \right. \kern-\nulldelimiterspace} {Z_{l} }}m_{h}$). It is clearly seen that the growth rate increases gradually with $\beta$. Thus, it can be fairly concluded that $\beta$ introduces a destabilizing influence on the system. An increase in ${{Z_{l} } \mathord{\left/ {\vphantom {{Z_{l} } {m_{l} }}} \right. \kern-\nulldelimiterspace} {m_{l} }}$ ($\sim \beta^{ - 1}$) gradually increases the elastic effects provided conjointly by the DES and LNS. Thus, decreasing $\beta$ should increase the NAW growth and vice-versa. But, a reverse is observed in both the NR (Fig. 3a) and UR (Fig. 3b) limits. It can be ascribed to ECP effects, dominating more in weakly coupled plasmas^24,34^. Higher the ${{Z_{l} } \mathord{\left/ {\vphantom {{Z_{l} } {m_{l} }}} \right. \kern-\nulldelimiterspace} {m_{l} }}$-value, higher is the ECP^24,34^ (due to higher $B_{l}$); and vice-versa. Thus, an enhanced ECP suppresses the instability growth (higher-${{Z_{l} } \mathord{\left/ {\vphantom {{Z_{l} } {m_{l} }}} \right. \kern-\nulldelimiterspace} {m_{l} }}$, lower-$\beta$); and vice-versa. Similarly, Fig. 4 depicts the colour spectral profiles of the growth rate as Fig. 2 for $\beta = 0.5$.

In a similar manner, Fig. 5 shows the same as Fig. 1, but for different values of the Coriolis rotation force. The distinct coloured lines clearly indicate that an increase in the Coriolis force results in an enhanced growth rate, in both the NR (Fig. 5a), and UR (Fig. 5b) limits. The Coriolis rotation destabilizes the system subject to the conjoint action of the concurrent effects of the considered factors simultaneously. The physical insight behind this is grounded on the fact that, greater the mass of the system, greater is the angular momentum, thereby leading to a higher degree of the Coriolis rotation. It is a well established dynamical reality in the diversified astrocosmical scenarios that heavier objects gravitationally collapse faster, and vice-versa. It hereby enables us to infer that the Coriolis rotational force plays an active role in the destabilization process of the system against the non-local long-range gravity. Lastly, Fig. 6 depicts almost the same features as Fig. 4, but for $C_{F}^{*} = 400$. It is noted that there exists some minor quantitative disparities ascribable to the parametric domains under analysis.

The obtained results, mainly on the Coriolis rotational role as a destabilizing agency, are fairly correlative and consistent with the previous astronomical findings on the gyratory compact astroobjects, as widely evident in the literature^28^. In fact, it has been practically found in the case of a white dwarf stars, like SS Cygni, CM Del, and so forth that its rotational speed fairly increases during the unstable outburst phase^32^, which reliably hints at the concretized accuracy of our proposed model analysis depicting rotation-induced destabilizing effects in such astrocompact circumstances.

The above analysis is restricted to the excited wave instability features just in the core and mantle of a rapidly rotating collapsing white dwarf stellar configuration, where the dominance of the three considered species (DES, LNS, HNS) indeed prevails^11–13^. The crust and atmosphere of the white dwarfs consist of alkali metals, mainly lithium (Li) and potassium (K)^38^, where our analysis would not be so appropriate to apply. It may be pertinent to add furthermore that the composition of the crust and atmosphere of degenerate white dwarfs can similarly be mapped to that of rocky planets, such as the Earth, Mars, and so forth^38^. Thus, the main limitation of our GH model-based study is the fact that the model analysis cannot be applied to the classical crust and atmosphere of a white dwarf star due to the postulated compositional disparity. Besides, the adopted idealized consideration of a spherically symmetric geometry with the polar and azimuthal wave-kinetic aspects completely ignored gives a clear indication for the future scope of a judicious model refinement in this direction. A further extensive applicability of the analysis, despite the above facts and faults, may also be relevant in the viscous evolution of white dwarf merger remnants and associated complex wave dynamics^31^.

## Conclusions

The presented analysis puts forward a theoretic model formulation to study the excitation and propagation dynamics of the NAWs in a compact astrophysical fluid system. The model is founded in a GH fabric practically resembling white dwarf interior environs. It considers a three-component plasma system composed of HNS, LNS and tiny quantum DES. It is interestingly under the concurrent action of the Bohm potential, Coriolis rotational force, ECP, self-gravity, and viscoelasticity. A standard normal spherical mode analysis over the perturbed DQP system yields a generalized dispersion relation (septic). It highlights the explicit dependency of various atypical parametric constants on the diversified equilibrium plasma properties. A numerical illustrative platform is provided to explore the multiparametric influential dependencies of the DQP fluctuation dynamics in detail. It presents different relevant two-dimensional growth-damping profiles (Figs. 1, 3, 5) and the corresponding colourspectral profiles (Figs. 2, 4, 6) with some minor quantitative differences in the NR limits and UR limits of the astrocosmic relevance.

It may be noteworthy that, Figs. 2, 4 and 6 as discussed above, are the colour spectral profiles obtained by changing the Matlab camera’s line of sight (i.e., orientation or projection) of the three-dimensional surface plots (with the wavenumber, distance, growth rate taken in three mutually independent perpendicular axes with a common origin). The actual three-dimensional surfaces are developed methodologically by executing the full numerical simulation of the generalized linear dispersion relation (septic in degree), given by Eq. (34), which is reduced in the low-frequency regime as Eq. (43), in the real platform of the Matlab programming. More technically, these three-dimensional figures are developed with the azimuthal and the elevation angles set equal to 0 and 90, respectively. Against this backdrop, it is already evident that Figs. 1, 3 and 5 are simply the two-dimensional spectral profiles obtained by the same dispersion analysis (with the wavenumber and growth rate taken in two independent perpendicular axes with a common origin).

The main conclusions drawn from this study include the fact that, in both the NR and UR limits, the charge density ratio of the heavy-to-light nuclear species ($\mu$) introduces a stabilizing influence on the system (Fig. 1). The charge-to-mass coupling parameter ($\beta$) destabilizes the system (Fig. 3). It can be further inferred from the proposed model analysis that the Coriolis rotation destabilizes the system (Fig. 5). The physical insights responsible behind are concisely illuminated in the relevant perspectives. It is substantiated fairly by the observed astronomical data^27,28,32^, which, reinforcingly, hint at the same Coriolis rotational effects, as investigated here.

White dwarfs are extremely compact astrophysical objects where the gravitational attraction is balanced by the non-thermal degenerate electronic pressure. Thus, degenerate electronic pressure plays a significant role throughout the life of a white dwarf star. The effect of viscoelastic dissipation is mainly visible for strongly coupled HNS in the parameter space defined by $0 < \tau_{m} < \infty$ ^37^. Also, for a rapidly rotating contracting white dwarf star approaching collapse, material in the envelope is shed when ${{v_{c}^{2} } \mathord{\left/ {\vphantom {{v_{c}^{2} } 2}} \right. \kern-\nulldelimiterspace} 2}\, = \,{{\left( {GM^{*} M_{\Theta } } \right)} \mathord{\left/ {\vphantom {{\left( {GM^{*} M_{\Theta } } \right)} {R_{c} }}} \right. \kern-\nulldelimiterspace} {R_{c} }}$; where, $M^{*}$ is the normalized mass of the star on the $M_{\Theta }$-scale, $R_{c}$ is the equatorial radius of the white dwarf star, and $v_{c}$ is the equatorial velocity^30^. At this stage of collapse, the material in rapidly rotating white dwarf stars is highly viscoelastic^30^. A significant fraction of mass of white dwarf merger remnants is initially supported by rotation. Post merger viscous phase causes detonation of the helium (He) envelope in white dwarf mergers^31^, thereby acting as potential triggering agents of Type-Ia supernovae. Thus, the obtained results may prove to be beneficial in understanding the diversified wave features in astrophysical compact objects, interiors, and correlated surroundings, especially white dwarfs, where the effects of viscoelastic dissipation, degenerate electron pressure, and strongly coupled inertial HNS play an important role.

It has been reported that there exist a rich plethora of more than hundred oscillation (pulsation) modes, both in pre-white dwarf stars, such as PG1159-035^13^, and in variable white dwarf stars, such as GD-358^13^. It has left behind an interesting scope for the future discovery of different collective waves, instabilities, and their saturation structures in such plasma media, with the proposed NAWs and propagatory dynamics as their special cases in extreme conditions as proposed herein. Lastly, it is reiterated that our results may have concrete and promising applications in understanding the evolution, excitation, and propagation dynamics of the NAWs and similar normal acoustic modes widely supported in compact correlated astroobjects and their interiors, such as white dwarfs, brown dwarfs, red dwarfs, neutron stars, etc.

## Supplementary Information

Supplementary Information 1.

Supplementary Information 2.

Supplementary Information 3.

Supplementary Information 4.
