# Supplementary material for: Propagatory dynamics of nucleus-acoustic waves excited in gyrogravitating degenerate quantum plasmas electrostatically confined in curved geometry
Source: Sci Rep. 2021 Sep 27;11:19126. doi: 10.1038/s41598-021-98543-2 (PMC8476626; doi:10.1038/s41598-021-98543-2)
Supplement: Supplementary file 1 — Supplementary Information 1. [file 41598_2021_98543_MOESM1_ESM.docx]

**Appendix-A: Adopted abbreviation scheme**

| **S. No.** | **Abbreviation** | **Expanded terminology** |
| --- | --- | --- |
| 1. | NAW | Nucleus-acoustic wave |
| 2. | DQP | Degenerate quantum plasma |
| 3. | HNS | Heavy nuclear species |
| 4. | LNS | Lighter nuclear species |
| 5. | DES | Degenerate electronic species |
| 6. | ECP | Electrostatic confinement pressure |
| 7. | NR | Non-relativistic |
| 8. | UR | Ultra-relativistic |
| 9. | K-dV | Korteweg-de Vries |
| 10. | m-KdV | Modified Korteweg-de Vries |
| 11. | NLS | Nonlinear Schrodinger |
| 12. | ZKB | Zakharov-Kuznetsov-Burgers |
| 13. | IAW | Ion-acoustic wave |
| 14. | GH | Generalized hydrodynamic |
| 15. | EM | Electromagnetic |
