# Supplementary material for: Propagatory dynamics of nucleus-acoustic waves excited in gyrogravitating degenerate quantum plasmas electrostatically confined in curved geometry
Source: Sci Rep. 2021 Sep 27;11:19126. doi: 10.1038/s41598-021-98543-2 (PMC8476626; doi:10.1038/s41598-021-98543-2)
Supplement: Supplementary file 2 — Supplementary Information 2. [file 41598_2021_98543_MOESM2_ESM.docx]

**Appendix-B: Adopted normalization scheme**

| **S. No.** | **Unnormalized parameter** | **Normalizing parameter** | **Normalized parameter** |
| --- | --- | --- | --- |
| 1. | Distance (*r*) | LNS Debye length ($\lambda_{Dl}$) | *ξ* |
| 2. | Time (*t*) | LNS oscillation time scale (*t_pl_*) | *τ* |
| 3. | Viscoelastic relaxation time (*τ_m_*) | LNS oscillation time scale (*t_pl_*) | $\tau_{m}^{*}$ |
| 4. | Population density (*n_s_* , s = e, l, h ) | Equilibrium number density (*n_s0_*) | *N_s_* |
| 5. | Velocity (*u_s_* , s= e, l, h) | LNS transit speed (*C_l_*) | *M_s_* |
| 6. | Polar rotational velocity (*v_θ_*) | LNS transit speed (*C_l_*) | $v_{\theta}^{*}$ |
| 7. | Azimuthal rotational frequency ($\Omega$*_ϕ_*) | LNS oscillation frequency (*ω_pl_*) | $\Omega_{\phi}^{*}$ |
| 8. | Temperature (*T*) | Ratio of electronic energy to Boltzmann constant (${m_{e}c^{2}}/{k_{B}}$) | *T^*^* |
| 9. | Polytropic constant (*K*) | ${{(m}_{e}c^{2}}/{n_{e0}^{\gamma_{e}-1}}$) | $K_{e}^{ʹ}$ |
| 10. | Effective generalized viscosity (*χ*) | ${(m}_{h}n_{h0}C_{l}\lambda_{Dl})$ | *χ* ^*^ |
| 11. | Proportionality constant of ECP for LNS (*B*) | Ratio of electronic energy to LNS equilibrium concentration (${m_{e}c^{2}}/{n_{l0}}$) | *B^*^* |
| 12. | Proportionality constant of ECP for HNS (*C*) | Ratio of electronic energy to HNS equilibrium density (${m_{e}c^{2}}/{n_{h0}}$) | *C^*^* |
| 13. | Electrostatic  potential ($\phi_{E}$) | Electronic kinetic potential (${m_{e}c^{2}}/e$) | *Φ_E_* |
| 14. | Gravitational potential (ψ) | Square of LNS transit speed ($C_{l}^{2}$) | *Ψ* |
