# Supplementary material for: Propagatory dynamics of nucleus-acoustic waves excited in gyrogravitating degenerate quantum plasmas electrostatically confined in curved geometry
Source: Sci Rep. 2021 Sep 27;11:19126. doi: 10.1038/s41598-021-98543-2 (PMC8476626; doi:10.1038/s41598-021-98543-2)
Supplement: Supplementary file 3 — Supplementary Information 3. [file 41598_2021_98543_MOESM3_ESM.docx]

**Appendix-C: IAWs vs. NAWs**

| **S. No.** | **Item** | **IAWs [Source]** | **NAWs [Source]** |
| --- | --- | --- | --- |
| 1. | Origin | Classical [31] | Quantum mechanical [7, 8] |
| 2. | Electron statistics | Boltzmann (classical) [31] | Fermi-Dirac (quantum) [3] |
| 3. | Restoring force | Electron thermal pressure (classical) [31] | Electron degeneracy pressure (quantum-mechanical) [1, 2, 7, 8] |
| 4. | Heavier species | Ions as inertial species [31] | Nuclei as inertial species [1, 2, 7, 8] |
| 5. | Existence at$T_{e}\sim0 K$ | Not possible (since no electron thermal pressure) [31] | Possible (since non-zero electron degeneracy pressure) [16] |
| 6. | Modal type | Compression and rarefaction of ions (longitudinal) [31] | Compression and rarefaction of nuclei (longitudinal) [1, 2] |
| 7. | Effect of rotation | Decreases energy of IA solitary wave [32] | Increases the growth rate of the NAW  [current work] |
| 8. | Comparison with normal sound mode | Result of heterogeneous classical coupling between electrons and ions (unlike the same species for usual sounds) [31] | Result of heterogeneous quantum coupling between DES, HNS, and LNS (unlike the same species for usual sounds) [1, 2, 7, 8 ] |
| 9. | Main influencing factors | Thermal motion of electrons [31] | Degeneracy pressure of electrons [7] |
| 10. | Degree of dispersion relation | 4 [32] | 7 (in this case) |
| 11. | Existence | Plasmas where $T_{i}\ll T_{e}$ [31] | Dense plasmas ( $T_{e}\sim0 K$, electron degeneracy pressure dominant) [16] |
