# Supplementary material for: Propagatory dynamics of nucleus-acoustic waves excited in gyrogravitating degenerate quantum plasmas electrostatically confined in curved geometry
Source: Sci Rep. 2021 Sep 27;11:19126. doi: 10.1038/s41598-021-98543-2 (PMC8476626; doi:10.1038/s41598-021-98543-2)
Supplement: Supplementary file 4 — Supplementary Information 4. [file 41598_2021_98543_MOESM4_ESM.docx]

**Appendix-D:**

After introducing perturbations and linearizing Eqs. (12)-(19), we get

(A) Linearized DES equations:

$\frac{\partial N_{e1}}{\partial\tau} + \frac{\partial M_{e1}}{\partial\xi} + \left(\frac{2}{\xi} \right)M_{e1} = 0 ,$ (D-1)

(D-2)

(B) Linearized LNS equations:

$\frac{\partial N_{l1}}{\partial\tau} + \frac{\partial M_{l1}}{\partial\xi} + \left(\frac{2}{\xi}\right) M_{l1} = 0 ,$ (D-3)

$\frac{\partial M_{l1}}{\partial\tau} + \frac{\partial\Phi_{E1}}{\partial\xi} + \frac{\partial\Psi_{1}}{\partial\xi} + A'_{l}\frac{\partial N_{l1}}{\partial\xi} \left( T^{*} + 2B_{l}^{*}\right) = 0 ,$ (D-4)

(C) Linearized HNS equations:

$\frac{\partial N_{h1}}{\partial\tau} + \frac{\partial M_{h1}}{\partial\xi} + \left(\frac{2}{\xi}\right) M_{h1} = 0 ,$ (D-5)

(D-6)

(D) Linearized coupling electrostatic Poisson equation:

$\frac{\partial^{2}\Phi_{E1}}{\partial\xi^{2}} + \left(\frac{2}{\xi} \right)\frac{\partial\Phi_{E1}}{\partial\xi} = \left(1+\mu\right) N_{e1} - N_{l1} - \mu N_{h1} ,$ (D-7)

(E) Linearized coupling self-gravitational Poisson equation:

$\frac{\partial^{2}\Psi_{1}}{\partial\xi^{2}} + \left(\frac{2}{\xi}\right) \frac{\partial\Psi_{1}}{\partial\xi} = \sigma\left(N_{l1} + \frac{\mu}{\beta} N_{h1}\right) .$ (D-8)

The above equations evolving in the coordination space $\left(\xi,\tau\right)$ are now transformed into the Fourier space $\left(\Omega,k^{*}\right)$. The transformation technique is defined by Eq. (20) with the geometric curvature effects. In the transformed wave space, we get the linear spatial and temporal operators get transformed as $\partial/\partial\xi\to\left( ik^{*}- 1/\xi\right)$ and $\partial/\partial\tau\to\left(-i \Omega\right)$, respectively. The perturbed quantities derived from the above equations here are respectively given by Eqs. (24)-(33) in the main text.
